# Identification of Hub Genes Associated With Clear Cell Renal Cell Carcinoma by Integrated Bioinformatics Analysis

**DOI:** 10.3389/fonc.2021.726655

**Published:** 2021-09-30

**Authors:** Hao Huang, Ling Zhu, Chao Huang, Yi Dong, Liangliang Fan, Lijian Tao, Zhangzhe Peng, Rong Xiang

**Affiliations:** ^1^ Department of Nephrology, Xiangya Hospital Central South University, Changsha, China; ^2^ Department of Cell Biology, School of Life Sciences, Central South University, Changsha, China; ^3^ Hunan Key Laboratory of Organ Fibrosis, Central South University, Changsha, China; ^4^ Department of Otolaryngology-Head and Neck Surgery, Second Xiangya Hospital Central South University, Changsha, China

**Keywords:** clear cell renal cell carcinoma, weighted gene co-expression network analysis, differentially expressed genes, prognostic genes, single-cell analysis

## Abstract

**Background:**

Clear cell renal cell carcinoma (ccRCC) is a common genitourinary cancer type with a high mortality rate. Due to a diverse range of biochemical alterations and a high level of tumor heterogeneity, it is crucial to select highly validated prognostic biomarkers to be able to identify subtypes of ccRCC early and apply precision medicine approaches.

**Methods:**

Transcriptome data of ccRCC and clinical traits of patients were obtained from the GSE126964 dataset of Gene Expression Omnibus and The Cancer Genome Atlas Kidney Renal Clear Cell Carcinoma (TCGA-KIRC) database. Weighted gene co-expression network analysis (WGCNA) and differentially expressed gene (DEG) screening were applied to detect common differentially co-expressed genes. Gene Ontology, Kyoto Encyclopedia of Genes and Genomes analysis, survival analysis, prognostic model establishment, and gene set enrichment analysis were also performed. Immunohistochemical analysis results of the expression levels of prognostic genes were obtained from The Human Protein Atlas. Single-gene RNA sequencing data were obtained from the GSE131685 and GSE171306 datasets.

**Results:**

In the present study, a total of 2,492 DEGs identified between ccRCC and healthy controls were filtered, revealing 1,300 upregulated genes and 1,192 downregulated genes. Using WGCNA, the turquoise module was identified to be closely associated with ccRCC. Hub genes were identified using the maximal clique centrality algorithm. After having intersected the hub genes and the DEGs in GSE126964 and TCGA-KIRC dataset, and after performing univariate, least absolute shrinkage and selection operator, and multivariate Cox regression analyses, *ALDOB*, *EFHD1*, and *ESRRG* were identified as significant prognostic factors in patients diagnosed with ccRCC. Single-gene RNA sequencing analysis revealed the expression profile of *ALDOB*, *EFHD1*, and *ESRRG* in different cell types of ccRCC.

**Conclusions:**

The present results demonstrated that *ALDOB*, *EFHD1*, and *ESRRG* may act as potential targets for medical therapy and could serve as diagnostic biomarkers for ccRCC.

## Introduction

Renal cell carcinoma (RCC) is one of the most common genitourinary cancer types worldwide, and it has a number of heterogeneous histological subtypes, with clear cell RCC (ccRCC) accounting for ~85% of all cases (1). In total, 431,288 new patients were diagnosed with renal cancer, and 179,368 of these patients succumbed to the disease worldwide in 2020 (2). ccRCC is not susceptible to chemoradiotherapy (3). Although ccRCC is curable at an early localized stage by partial or total surgical nephrectomy, advanced or metastatic ccRCC remains a clinical challenge (4). Over the past years, antiangiogenic treatment, inhibitors of the mammalian target of rapamycin (mTOR) pathway, or immune checkpoint inhibition therapy have considerably evolved (5). However, due to diverse biochemical alterations and a high level of tumor heterogeneity, it is important to select highly validated prognostic biomarkers to identify subtypes of ccRCC early and apply precision medicine approaches (6).

The molecular mechanism of ccRCC is characterized by genetic diversity and chromosomal complexity. Loss of the heterozygosity of chromosome 3p, where the von Hippel–Lindau (*VHL*) gene is located, is found in over 90% of ccRCC cases, and it is considered the critical genetic event (7–9). A loss-of-function mutation in the *VHL* gene induces the aberrant regulation of a number of *VHL*-mediated targets, pathways, and processes, which is a significant step in the development of ccRCC (10, 11). The VHL protein, as an E3 ubiquitin ligase, is notably involved in the ubiquitylation of the prolyl hydroxylated transcription factors, hypoxia-inducible factor 1 α (HIF1 α) and HIF2 α, under normoxic conditions. HIF1 α and HIF2 α have an important role in the regulation of angiogenesis, erythropoiesis, glycolysis, and apoptosis (12–14). Moreover, next-generation sequencing technologies have provided evidence that *PBRM1*, *SETD2*, or *BAP1* mutations are the drivers of tumor evolution (15, 16). Although the molecular features of ccRCC have been increasingly defined by previous studies (17–19), there remain numerous subtypes of ccRCC the pathogenic mechanisms of which have yet to be clearly determined at the genetic and molecular levels. Thus, it is important to identify more additional disease-related genes.

Benefiting from the rapid development of genome sequencing technology, bioinformatics can be used to study gene expression profiles in order to examine the molecular mechanism of tumors and identify tumor-specific indicators. Weighted gene co-expression network analysis (WGCNA) was developed by Horvath and Zhang in 2005 (20). At present, WGCNA is becoming a powerful approach to detecting gene modules, exploring the correlation of the modules and phenotypes, and discovering hub genes that regulate critical biological processes (21, 22).

In the present study, a gene expression profile of ccRCC from the Gene Expression Omnibus (GEO) was downloaded. WGCNA and differentially expressed gene (DEG) screening were applied to detect common differentially co-expressed genes. Then, The Cancer Genome Atlas Kidney Renal Clear Cell Carcinoma (TCGA-KIRC) data were used to establish the prognostic model of ccRCC. Single-cell RNA sequencing (RNA-seq) data from GEO were used to verify the expression profile of the prognostic genes in different cell types. This study aimed not only to understand ccRCC pathogenesis but also to determine its molecular mechanisms and provide insights into novel therapeutic targets for drugs.

## Methods

### Data Collection and Single-Cell RNA Sequencing Data Processing

**Figure 1:**
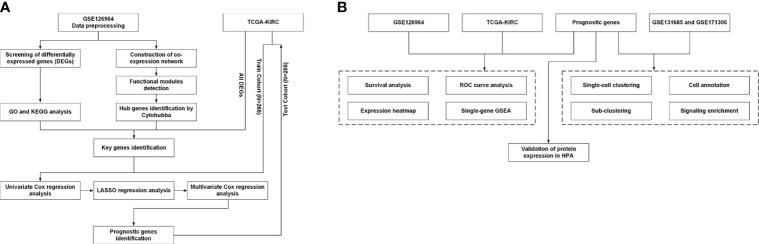
Workflow of the present study. **(A)** Identification workflow. **(B)** Verification workflow. DEG, differentially expressed gene; GO, Gene Ontology; KEGG, Kyoto Encyclopedia of Genes and Genomes; TCGA, The Cancer Genome Atlas; LASSO, least absolute shrinkage and selection operator; ROC, receiver operating characteristic; HPA, Human Protein Atlas; GSEA, gene set enrichment analysis.

The workflow for the current study is presented in **Figure 1**. Original data were collected from the GSE126964 dataset, which contained 55 ccRCC tumor tissues and 11 matched normal tissues (23). The GEO expression matrix was annotated with gene symbols using the information from the GPL20795 HiSeq X Ten platform file, as well as log_2_ transformed in R (version 4.0.4) and RStudio (version 1.2.5033) if necessary. Principal component analysis (PCA) was performed, and the outliers of GSM3619137 and GSM3619152 were excluded (**Figure S1**). In total, only 53 ccRCC sample and 11 normal sample data were used for subsequent analysis.

RNA-seq data of TCGA-KIRC and corresponding clinical information were obtained from TCGA (https://portal.gdc.cancer.gov/).

Single-cell RNA-seq data from GSE131685 and GSE171306 were downloaded through GEO website. R package “Seurat” (version 4.0.2) was used to process the data (24). Three healthy kidney samples from GSE131685 (25) and two ccRCC samples from GSE171306 (26) were merged for further analysis. The single-cell RNA-seq data processing was described previously (27). The cell clusters were annotated manually based on previous knowledge and information from literatures (28, 29). Expression profiling of the genes were depicted by heatmap and violin plot using the function “FeaturePlot” and “VlnPlot.”

### Differentially Expressed Gene Identification

The “limma” software package (version 3.48.0) (30) was used to conduct the DEG analysis between ccRCC and normal sample data from the GSE126964 dataset (30). An adjusted p-value <0.05 and a |log_2_fold change| of ≥2.0 were selected as the cutoff criteria. The volcano and heatmap plots were generated using ggplot2 (version 3.3.3) and pheatmap (version 1.0.12) packages, respectively. The DEGs of TCGA-KIRC dataset (https://portal.gdc.cancer.gov/) were obtained *via* Gene Expression Profiling Interactive Analysis (GEPIA2; http://gepia2.cancer-pku.cn/) (31) using the same aforementioned threshold.

### Gene Ontology Enrichment and Kyoto Encyclopedia of Genes and Genomes Analysis of Differentially Expressed Genes

The “clusterProfiler” (version 3.18.1) R package was used for GO and KEGG enrichment analyses (http://www.bioconductor.org/packages/release/bioc/html/clusterProfiler.html) (32). The three main processes in GO analysis are as follows: biological process (BP), molecular function (MF), and cellular component (CC). The p-value was conventionally set at 0.05. A circle plot was generated by “Goplot” R package (version 1.0.2).

### Weighted Gene Co-Expression Network Construction

The “WGCNA” package (version 1.70-3) of R (20) was used to construct the co-expression networks. Genes with mean counts of over 5 were selected. A total of 64 samples were used to calculate the Pearson’s correlation matrices. The matrices of adjacency were created based on the Pearson’s correlation matrices. Then, the clinical trait data were uploaded, and the scale independence and mean connectivity were estimated. Subsequently, the topological overlap measure (TOM) matrix, which was created from the adjacency matrix, was used to estimate the network’s connectivity property. A hierarchical clustering dendrogram of the TOM matrix was constructed using the average distance with a minimum size threshold of 50 to classify the similar gene expression profiles into different gene modules. Finally, similar gene modules were merged, with a threshold of 0.20.

### Co-Expression Network Construction and Hub Gene Identification

The Cytoscape software v3.7.2 was used to visualize the co-expression network in the turquoise module (33). The data were imported into Cytohubba, a Cytoscape plug-in for hub gene identification, and the maximal clique centrality (MCC) algorithm was used to calculate the scores of all nodes of the network. The top 30 nodes with the highest MCC scores were selected as the hub genes associated with ccRCC. The “real” key genes were identified as those intersecting between the top 30 nodes in turquoise module, DEGs from GSE126964 and DEGs from TCGA-KIRC.

### Identification and Verification of Prognostic Gene Signatures

Univariate Cox regression analysis was performed to screen the genes significantly associated with overall survival (OS) in the TCGA-KIRC dataset. The OS-related genes with p < 0.1 were included in the least absolute shrinkage and selection operator (LASSO) regression analysis by using the R package “glmnet” (version 4.1-2). Then, a multivariate Cox regression model analysis was performed to establish a Cox proportional hazards regression prognostic model. We used the following formula to calculate the risk score of each patient:


$$\text{Risk Score}=\sum_{i=1}^{n}\beta_{i}\times x_{i}$$

In this formula, *β* is coefficient and *x* is the expression level of each prognostic gene *i*. The samples were divided into a high-risk group and a low-risk group according to the median risk score of the training cohort from TCGA-KIRC. Receiver operating characteristic (ROC) analysis and Kaplan–Meier analysis were conducted between the high-risk group and the low-risk group.

### Validation of the Protein Expression Levels of Prognostic Genes in the Human Protein Atlas Database

The Human Protein Atlas (HPA) is a database that aims to map all the human proteins in cells, tissues, and organs using an integration of various omics technologies (https://www.proteinatlas.org/). We also verified the protein expression levels of the survival-related hub genes based on immunohistochemistry using the HPA database.

### Gene Set Enrichment Analysis of Prognostic Genes

Gene set enrichment analysis (GSEA) was also used to detect the potential molecular mechanisms of the prognostic genes. Enriched terms predicted to be associated with the KEGG pathway in c2.cp.v7.2.symbols.gmt were screened by GSEA. Images were generated by “ggplot2” (version 3.3.3) package. The p-value of <0.05 was considered statistically significant.

### Prognostic Gene Expression Profiles

The prognostic gene expression profiles were obtained from the GTEx Portal (https://gtexportal.org/home/).

## Results

### Differentially Expressed Gene Screening

**Figure 2:**
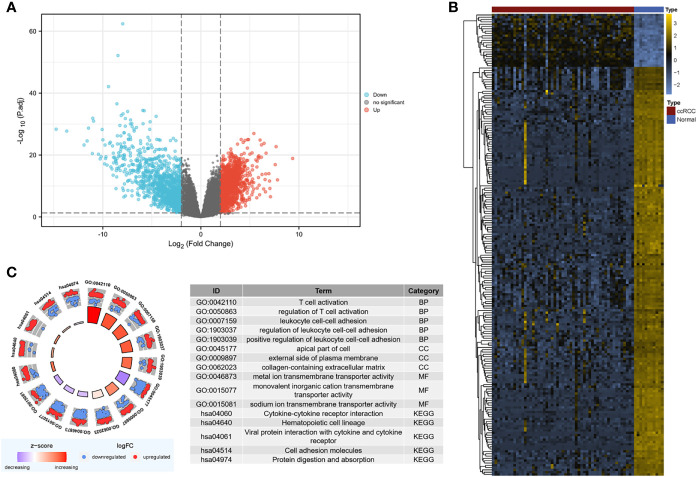
Screening for DEGs. **(A)** Volcano map of DEGs between ccRCC and normal samples in the GSE126964 dataset. The red plots in the volcano represent upregulated genes, and the blue points represent downregulated genes. **(B)** Heatmap of the 200 selected DEGs. The color in heatmaps from blue to yellow shows the progression from low expression to high expression, respectively. **(C)** GO and KEGG analyses of the DEGs. The outer circle shows the scatter plot of the assigned gene log_2_fold change for all terms: red points show genes that exhibited increased expression, whereas the blue points represent genes that exhibited decreased expression. The inner circle indicates the Z-score value and the number of genes. Red represents a higher z-score value, and purple represents a lower Z-score value. DEG, differentially expressed gene; BP, biological process; CC, cell component; MF, molecular function; GO, Gene Ontology; KEGG, Kyoto Encyclopedia of Genes and Genomes; ccRCC, clear cell renal cell carcinoma.

The “limma” package was utilized to analyze DEGs in the GSE126964 dataset, with the threshold of |log_2_(fold-change)|>2.0 and adjusted p < 0.05. A total of 2,492 DEGs between ccRCC and normal control samples were filtered, revealing 1,300 upregulated genes and 1,192 downregulated genes (**Figures 2A, B** **)**.

The DEGs were mostly enriched in “T cell activation,” “leukocyte cell–cell adhesion,” “apical part of cell,” “external side of plasma membrane,” “collagen-containing extracellular matrix (ECM),” and “ion transmembrane transporter activity” in the GO analysis (**Figure 2C**). In the KEGG analysis, DEGs were enriched in “cytokine–cytokine receptor interaction,” “hematopoietic cell lineage,” “viral protein interaction with cytokine and cytokine receptor,” “cell adhesion molecules,” and “protein digestion and absorption” (**Figure 2C**).

We also evaluated the metabolic shift between ccRCC tissues and normal control tissues in the GSE126964 dataset. Similar to the finding of Clark et al. (19), glycolysis-associated genes were found to be significantly upregulated, and most oxidative phosphorylation (OXPHOS) and tricarboxylic acid (TCA) cycle-associated genes were significantly downregulated in the GSE126964 dataset (**Figure S2**).

### Weighted Co-Expression Network Construction and Analysis

**Figure 3:**
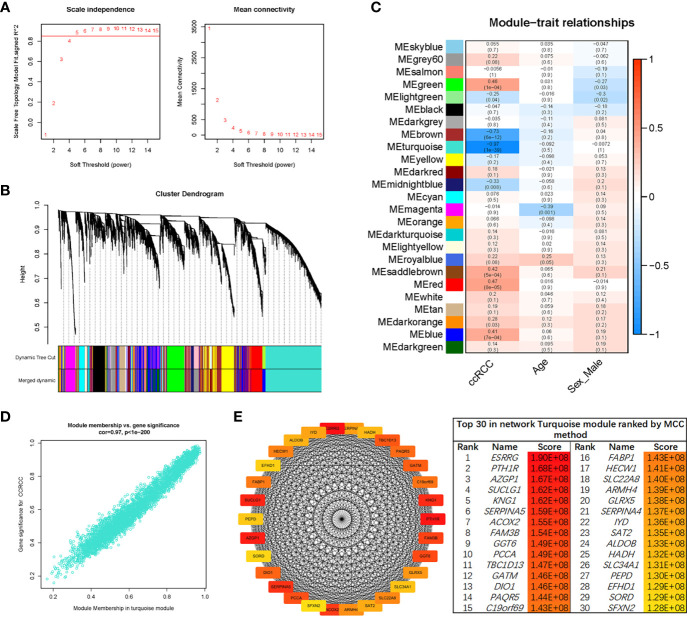
WGCNA of the ccRCC samples. **(A)** Analysis of the network topology for various soft-thresholding powers. The left plot shows the scale-free fit index (y-axis) as a function of the soft-thresholding power (x-axis). The horizontal red line shows x-axis = 0.85. The right plot displays the mean connectivity (degree, y-axis) as a function of the soft-thresholding power (x-axis). The power was set as 5 for further analysis. **(B)** Hierarchical cluster analysis was conducted to detect co-expression clusters with corresponding color assignments. Each color represents a module in the constructed gene co-expression network, as assessed *via* WGCNA. **(C)** Module–trait relationships. Each row represents a color module, and every column represents a clinical trait. Each cell contains the corresponding correlation and p-value. **(D)** A scatter plot of GS for ccRCC *vs.* the MM in the turquoise module. **(E)** Identification of hub genes using the MCC method. Genes with the top 30 MCC values were colored red to yellow. Red refers to a relatively large MCC value, and yellow refers to relatively smaller MCC values. WGCNA, weighted gene co-expression network analysis; ccRCC, clear cell renal cell carcinoma; GS, gene significance; MM, module membership; MCC, maximal clique centrality.

The sample clustering dendrograms of the ccRCC and normal samples are shown in **Figure S3A**. The soft-power threshold *β* was selected as 5 to ensure that both the scale-free topology model fit index (R^2^) and mean connectivity reached steady status (**Figure 3A**). Then, gene modules were detected based on the TOM matrix. A total of 25 modules were identified *via* average linkage hierarchical clustering, and each module was represented by a different color (**Figure 3B**). Among the modules, the turquoise module had the highest correlation with ccRCC traits (*r* = -0.97, p = 1e-39) (**Figure 3C**). A set of 400 selected genes were identified for the network heatmap construction (**Figure S3B**).

### Identification of Key Genes

An intramodular analysis of gene significance (GS) and module membership (MM) of the genes in the module turquoise was subsequently conducted. A high correlation coefficient of GS and MM was found in the turquoise module (cor = 0.97, p < 1e-200) (**Figure 3D**). The co-expression network of the turquoise module was constructed using Cytoscape software. Then, the module net was analyzed with the “Cytohubba” plug-in, and a network of the top 30 hub genes was constructed using the MCC algorithm (**Figure 3E**).

**Figure 4:**
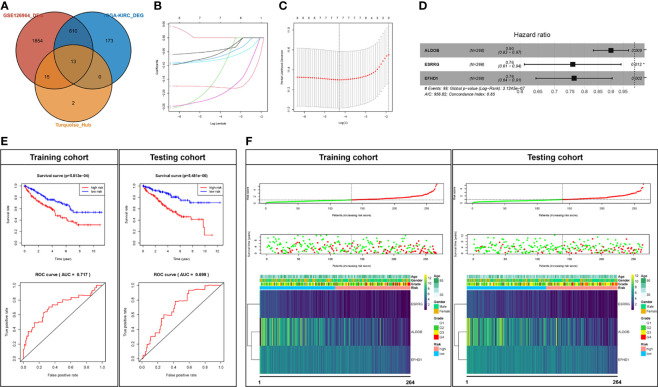
Prognostic analysis of the key genes. **(A)** Key genes belonging to both the hub genes and DEGs of the GSE126964 and TCGA-KIRC datasets. **(B**, **C)** LASSO regression complexity was controlled by lambda using the glmnet R package. **(D)** The multivariate analysis of risk factors in ccRCC. **(E)** Overall survival and ROC analysis between high-risk score and low-risk score groups in the training cohort and testing cohort. **(F)** The overall survival stratified by the high- and low-risk score groups was plotted for the training cohort and testing cohort. Detailed risk scores, survival information, and heat maps of gene expression are also included for each dataset. ^*^p < 0.05; ^**^p < 0.01; DEG, differentially expressed gene; TCGA-KIRC, The Cancer Genome Atlas Kidney Renal Clear Cell Carcinoma; ROC, receiver operating characteristic; AUC, area under the curve; ccRCC, clear cell renal cell carcinoma; LASSO, least absolute shrinkage and selection operator.

In order to identify the “real” key genes, we then obtained 796 DEGs, using a cohort of KIRC, from TCGA *via* GEPIA2, with the same threshold values. After comparing the DEGs in the GSE126964 dataset, TCGA-KIRC data and the top 30 hub genes from the turquoise module, a set of 13 key genes was identified (**Figure 4A**).

### Validation of Key Genes *via* Survival Analysis

We randomly divided the patients in TCGA-KIRC into two cohorts, a training cohort (*N* = 266) and a testing cohort (*N* = 266). The univariate Cox regression analyses of 13 key genes with regard to OS of samples from the training cohort were performed (**Table 1**). Eight genes with p < 0.1 (*GGT6*, *SLC22A8*, *FAM3B*, *PTH1R*, *ALDOB*, *ESRRG*, *SLC34A1*, and *EFHD1*) were included in LASSO analysis (**Figures 4B, C** **)**. Following the cross validation, seven genes achieved the minimum partial likelihood deviance. Then, we performed a multivariate Cox regression with these seven genes (*GGT6*, *FAM3B*, *PTH1R*, *ALDOB*, *ESRRG*, *SLC34A1*, and *EFHD1*) as covariants. We finally got three genes, including *ALDOB*, *ESRRG*, and *EFHD1* without collinearity, and each of them could be an independent prognostic marker for ccRCC (**Figure 4D**). A prognostic model based on the three genes was established. The risk score for each individual patient was calculated with the following formula: risk score = (-0.105197) * *ALDOB* + (-0.275676) * *ESRRG* + (-0.269554) * *EFHD1*.

**Table 1:** Univariate Cox regression analysis in the train cohort.

Characteristics	HR	95% CI	*p*-value
*GGT6* (High *vs.* Low)^#^	0.702	0.535-0.922	0.011*
*KNG1* (High *vs.* Low)	0.995	0.856-1.157	0.952
*DIO1* (High *vs.* Low)	0.938	0.744-1.181	0.584
*SLC22A8* (High *vs.* Low)	0.706	0.531-0.937	0.016*
*FAM3B* (High *vs.* Low)	0.738	0.519-1.049	0.090
*SERPINA5* (High *vs.* Low)	1.047	0.932-1.175	0.440
*FABP1* (High *vs.* Low)	0.920	0.776-1.091	0.340
*PTH1R* (High *vs.* Low)	0.795	0.693-0.911	0.001*
*ARMH4* (High *vs.* Low)	0.783	0.570-1.075	0.131
*ALDOB* (High *vs.* Low)	0.873	0.809-0.942	<0.001***
*ESRRG* (High *vs.* Low)	0.685	0.557-0.843	<0.001***
*SLC34A1* (High *vs.* Low)	0.825	0.700-0.973	0.022*
*EFHD1* (High *vs.* Low)	0.700	0.594-0.825	<0.001***

HR, hazard ratio; CI, confidence interval; ^#^Samples were classified into high and low gene expression according to a cut-off of 50%; *p < 0.05; ***p < 0.001.

Then, the Kaplan–Meier analysis was performed. As shown in **Figure 4E**, the survival rate of patients in the high-risk group was significantly lower than that in the low-risk group in either training cohort (p < 5.81e-4) or testing cohort (p < 5.48e-20). The ROC curve was then used to evaluate the accuracy of the survival analysis. The areas under the curves (AUCs) were 0.717 and 0.699 in the training cohort and testing cohort, respectively (**Figure 4E**), which indicate that the prediction effect was good. We also plotted the distribution of risk scores in patients with ccRCC and the correlation between survival time and risk scores in the training cohort and testing cohort (**Figure 4F**). In addition, all of the three genes (*ALDOB*, *ESRRG*, and *EFHD1*) were significantly downregulated and associated with poor pathologic stages in the training cohort, testing cohort, or GSE126964 dataset (**Figure 4F** and **Figure S4**). Moreover, *ALDOB*, *ESRRG*, and *EFHD1* were highly expressed in renal tissues among the different normal tissues, which indicated a critical regulatory function of these genes in the normal kidney (**Figure S5**).

### Validation of Protein Expressions of Prognostic Genes

**Figure 5:**
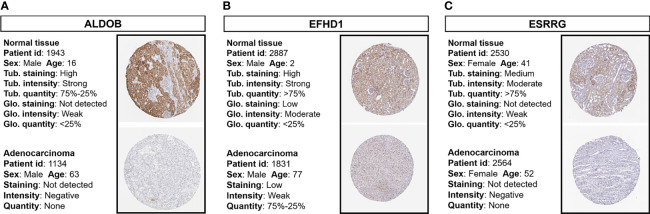
Immunohistochemistry staining of prognostic proteins based on the HPA. Protein expression levels of **(A)** ALDOB, **(B)** EFHD1, and **(C)** ESRRG in tumor tissues and normal tissues. HPA, Human Protein Atlas; Tub., tubules; Glo., glomeruli.

Immunohistochemistry staining results obtained from the HPA database revealed the protein expression levels of the key survival-related genes (**Figure 5**). The results showed the downregulation of ALDOB, EFHD1, and ESRRG protein in ccRCC samples compared with normal controls.

### Gene Set Enrichment Analysis of Prognostic Genes

**Figure 6:**
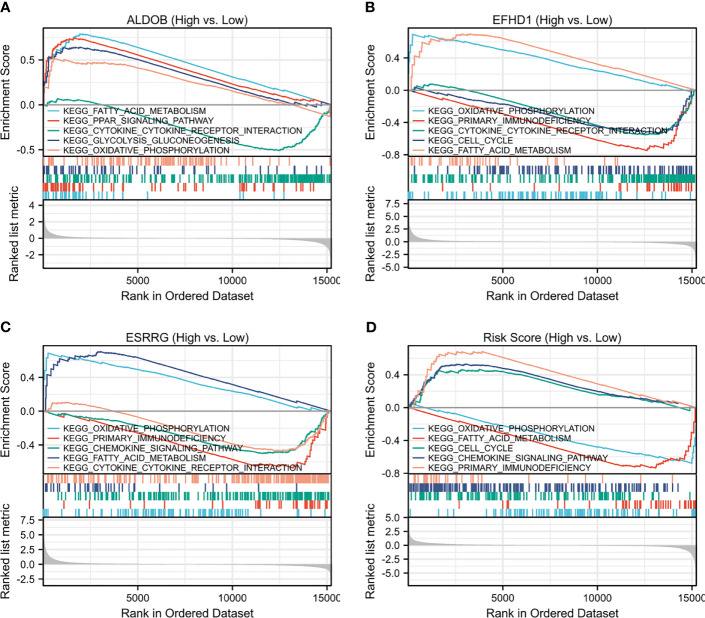
Single-gene GSEA of **(A)** ALDOB, **(B)** EFHD1, **(C)** ESRRG and **(D)** risk score based on TCGA-KIRC. GSEA, gene set enrichment analysis; KEGG, Kyoto Encyclopedia of Genes and Genomes; TCGA-KIRC, The Cancer Genome Atlas Kidney Renal Clear Cell Carcinoma.

GSEA was conducted to search the KEGG pathways in which the prognostic genes and risk scores were enriched in the samples with high expression or high-risk levels from TCGA-KIRC. “Oxidative phosphorylation” and “Fatty acid metabolism” pathways were enriched with low-risk score and high expression of *ALDOB*, *ESRRG*, and *EFHD1*, while immune-related pathways, including “Cytokine–cytokine receptor interaction,” “Chemokine signaling pathway,” and “Primary immunodeficiency” were significantly enriched with high-risk score and low expression of the prognostic genes (**Figures 6A–D**).

### Single-Cell Transcriptomic Context of the Prognostic Genes

**Figure 7:**
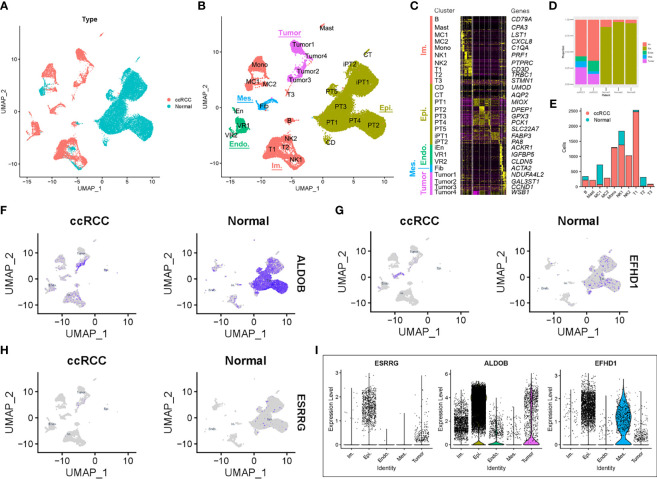
Prognostic expression profile based on single-cell sequencing analysis. **(A)** Composition and distribution of single cells from GSE131685 and GSE171306. **(B)** UMAP embedding of 34,371 single cells from three human normal kidneys and two ccRCC samples. Labels refer to 27 clusters identified. **(C)** Scaled gene expression of the top 10 specific genes in each cluster. Each column is the average expression of all cells in a cluster. **(D)** Composition of different cell types in the five single-cell RNA-seq samples. **(E)** Number of cells per immune cell type and clinical parameter. The expression profile of **(F)** *ALDOB*, **(G)** *EFHD1*, and **(H)** *ESRRG* for each cell and the **(I)** violin plot. UMAP, Uniform Manifold Approximation and Projection; ccRCC, clear cell renal cell carcinoma; Im., immune; Epi., epithelial; Endo., endothelial; Mes, mesenchymal; CD, collecting duct; CT, connecting tubule; iEn, injured endothelial cells; Fib, fibroblast; Mast, mast cell; MC, macrophage; Mono, monocyte; PT, proximal tubule; iPT, injured proximal tubule; VR, vasa recta.

To further verify the relationship among *ALDOB*, *ESRRG*, and *EFHD1* in ccRCC, single-cell RNA-seq data from GSE131685 and GSE171306 were employed (25, 26). After quality control, a total of 34,371 cells from two ccRCC samples and three normal kidney samples were profiled (**Figure 7A**). We identified 27 different cell clusters and five cell groups, including immune cells, epithelial cells, endothelial cells, mesenchymal cells, and tumor cells (**Figures 7B, C** **)**. Consistent with previous research (28), proximal tubular epithelial cells account for over 90% of a normal renal cortical sample, while in ccRCC, over 50% was accounted for immune cells and approximate 20% for tumor cells (**Figure 7D**). Except the clusters of macrophage 1 (MC1) and T cell 2 (T2), most kinds of immune cells were identified from ccRCC patients, which depicted a tumor immune microenvironment of ccRCC (**Figure 7E**). We also identified four tumor cell clusters. Analysis of KEGG pathway in tumor cells suggested the increased glycolysis gluconeogenesis, cancer, and focal adhesion-associated metabolism in ccRCC, while oxidative phosphorylation-associated pathways were negatively enriched with tumor cells (**Figures S6**, **S7**).

We then explored the expression profile of *ALDOB*, *ESRRG*, and *EFHD1* in different types of cells. Similar with results from TCGA-KIRC and GSE126964, the expression of *ALDOB*, *ESRRG*, and *EFHD1* were much lower in tumor cells than that in other intrinsic renal cells (**Figures 7F–I**).

## Discussion

ccRCC is a common genitourinary cancer with a high mortality rate (3). There is an urgent requirement to identify additional potential targets for drugs and biomarkers for early diagnosis of ccRCC. In the present study, a novel prognostic model based on three genes (*ALDOB*, *EFHD1*, and *ESRRG*) for ccRCC was established. *ALDOB*, *EFHD1*, and *ESRRG* were also identified as novel independent prognostic markers for ccRCC in different datasets *via* integrated bioinformatics analysis, including DEG analysis, WGCNA, and single-cell analysis.

### Metabolic Shift in Clear Cell Renal Cell Carcinoma

Metabolic disorder is a hallmark in different types of cancer, since sufficient energy and metabolite production are required for the malignant proliferation of cancer cells (34). Gebhard et al. (35) reported that ccRCC tissues were overloaded with glycogen and lipid compared with normal tissues, which suggested that the metabolism of lipids and glucose may be altered in ccRCC (36). In particular, a mutation in *VHL* is considered to be closely associated with metabolic reprogramming in ccRCC (37). Subsequent accumulation of HIF1 α leads to the expression of glucose transporter-1, thereby promoting cellular glucose uptake. In addition, it can induce lactate dehydrogenase, which promotes the conversion of pyruvate to lactate and switches energy production from the TCA to lactate fermentation (38). This phenomenon is widely known as the Warburg effect. Despite the well-known VHL–HIF axis, there are a number of altered levels of the biochemical enzymes, substrates, and metabolic intermediates or products that are involved in the metabolic reprogramming waiting to be discovered. Our analysis of the GSE126964 data and single-cell RNA-seq data from GSE131685 and GSE171306 supported the observations of the metabolic shift in ccRCC and demonstrated the upregulation of glycolysis-associated genes and downregulation of OXPHOS and TCA-associated genes at the transcriptome level (**Figure 6** and **Figures S2**, **S6**, **S7**). Although a metabolic shift is advantageous for tumor progression, altered metabolic pathways in ccRCC may also be exploited as therapeutic targets and may be an important future research direction.

### *ALDOB*, *EFHD1*, and *ESRRG* Can Be Novel Independent Prognostic Markers for Clear Cell Renal Cell Carcinoma

*ALDOB* encodes aldolase B, an enzyme that is expressed in the liver and kidneys and is involved in glycolysis process and fructolysis process. The function of which can cleave fructose-1,6-bisphosphate to yield glyceraldehyde and dihydroxyacetone phosphate (39). A research found that declined *ALDOB* expression was associated with multiple malignant characteristics of HCC and indicate a poor prognosis (40). Moreover, Bu et al. (41) shows that *ALDOB* upregulation is commonly found in the metastatic cell in liver during primary colon cancer proliferation by enhancing fructose metabolism and central carbon metabolism. A study by Wang et al. (42) found that the low expression of *ALDOB* is also important in ccRCC and predicts poor prognosis, which is consistent with our research results. It leads to a high level of fructose 1,6-bisphosphate (FBP) and protects ccRCC from oxidative stress (42). However, the mechanism and prognostic value of accumulated FBP remain unknown.

*EFHD1* encodes a mitochondrial inner membrane protein, acted as a calcium sensor for mitochondrial flash activation (43), and induced metabolic changes during the development of pro-/pre-B cells (44). A recent report suggested that EFHD1 may interact with β-actin for its involvement in the Ca^2+^-dependent regulation of mitochondrial morphology (45). *EFHD1* was significantly downregulated in both the GSE126964 and TCGA-KIRC cohorts (**Figure 4**) and may also have an impact on mitochondrial energy metabolism in ccRCC. However, the detailed mechanism of how EFHD1 regulates ccRCC pathogenesis is currently unknown.

*ESRRG* encodes a member of nuclear receptor superfamily of transcription factors and has been shown to be a tumor suppressor in different types of cancer (46–48). A study by Huang et al. (49) also identified *ESRRG* as a co-expressed DEG in different datasets of hypertension-related RCC. Moreover, an experimental study on the mechanism of *ESRRG* conducted by Nam et al. (50) demonstrated that ESRRG suppressed the migratory and invasive abilities of behaviors in RCC cells. Our analysis and previous studies have all suggested that lower *ESRRG* expression may be a reliable predictor of a poor clinical outcome.

### Hub Genes Including *ARMH4*, PTH1R, *SLC22A8*, and *SLC34A1* Were Correlated With Cancer

*ARMH4*, also named *C14ORF37*, encodes a protein that contains an armadillo-like helical domain. It has been shown that ARMH4 can interact with and inhibit the function of mTOR complex 2 kinase activity and function as a tumor suppressor in hematological malignancies, which is driven by interleukin 6 (IL-6)–signal transducer and activator of transcription 3 (STAT3) signaling pathways (51, 52). Wang et al. (53) also predicted that ARMH4 may act as a modulator for QKI, KH domain containing RNA binding, one of the key RNA-binding proteins shown in TCGA-KIRC dataset, and may change its splicing regulation in kidney cancer (53). Our analysis further confirmed the importance of *ARMH4* in ccRCC. However, the specific mechanism remains to be explored.

*PTH1R* encodes a G protein-coupled receptor of parathyroid hormone (PTH) and PTH-related protein and plays a central role in calcium homeostasis (54). Recently, the structure and dynamics of the active PTH1R have been shown by cryo-electron microscopy (55). Studies from different research groups have reported that the decreased expression of PTH1R was a poor prognosis factor in multiple types of cancer (56–59). Although PTH1R was found to be highly expressed in normal kidney samples (**Figure 5** and **Figure S4**), the detailed mechanism of PTH1R in renal function and ccRCC has yet to be fully elucidated and requires further study.

SLCs are a superfamily of membrane proteins responsible for the cellular uptake of a diverse range of substances. Among the SLCs, SLC22A8 and SLC34A1 both show kidney-specific expression. SLC22A8 is involved in the sodium-independent transport and excretion of organic anions, while SLC34A1 is a sodium-phosphate co-transporter that controls proximal tubule phosphate reabsorption (60, 61). Although defects in SLCs can lead to serious diseases (62–64), there is lack of research in cancer, especially ccRCC. Kang et al. (65) examined the expression patterns and prognostic values of SLCs in the development of ccRCC using different bioinformatics methods. These authors demonstrated that the low expression levels of a cluster of SLCs, including SLC34A1, were correlated with ccRCC progression and poor prognosis (65). Since ccRCC shows a prominent metabolic shift effect, the production and accumulation of metabolites are also different from those in normal tissues. Therefore, SLCs may be critical in ccRCC.

## Conclusion

Through a series of comprehensive bioinformatics analyses, including DEG screening, WGCNA, and single-cell analysis, a prognostic model based on *ALDOB*, *EFHD1*, and *ESRRG* was established, and these three genes were also identified as independent prognostic markers for ccRCC. The aforementioned prognostic genes have the potential to become therapeutic targets and biomarkers for ccRCC. However, these key survival-related genes should be tested in a large cohort of ccRCC cases and should be analyzed and validated in additional *in vivo* and *in vitro* experiments.

## Data Availability Statement

The original contributions presented in the study are included in the article/**Supplementary Material**. Further inquiries can be directed to the corresponding authors.

## Ethics Statement

The review board of the Xiangya Hospital of Central South University approved the present study.

## Author Contributions

ZP, RX, and LT conceived and directed the project. HH and LZ collected the data and information. HH, CH, YD, and LF analyzed and interpreted the data. HH wrote the manuscript with the help of all the other authors. All authors contributed to the article and approved the submitted version.

## Funding

This study was supported by the project funded by China Postdoctoral Science Foundation (2020TQ0363 and 2020M682598), the National Natural Science Foundation of China (82073918, 82090020, and 82090024), the Natural Science Foundation of Hunan, China (2021JJ40992), and the Fundamental Research Funds for the Central Universities of Central South University (2021zzts0078).

## Conflict of Interest

The authors declare that the research was conducted in the absence of any commercial or financial relationships that could be construed as a potential conflict of interest.

## Publisher’s Note

All claims expressed in this article are solely those of the authors and do not necessarily represent those of their affiliated organizations, or those of the publisher, the editors and the reviewers. Any product that may be evaluated in this article, or claim that may be made by its manufacturer, is not guaranteed or endorsed by the publisher.

## Supplementary Material

Supplementary Figure 1 PCA based on the whole gene list. PCA, principal component analysis.

Supplementary Figure 2 Schematic of metabolic pathways and their selected genes for glycolysis, TCA and electron transport chain, with the log_2_FC level between ccRCC and control samples in the GSE126964 dataset. **p*<0.05; ***p*<0.01; ****p*<0.001. FC, fold change; ccRCC, clear cell renal cell carcinoma; TCA, tricarboxylic acid cycle.

Supplementary Figure 3
**(A)** Sample clustering was conducted to detect outliers. All samples are located in the clusters and pass the cutoff thresholds after removing outliers. **(B)** Heatmap depicting the TOM of genes selected for weighted co-expression network analysis. The light color represents lower overlap, and red represents higher overlap. TOM, Topological Overlap Matrix.

Supplementary Figure 4 The overall survival stratified by the high and low-risk score groups was plotted for the GSE126964 dataset. Detailed risk scores, survival information and heat maps of gene expression are also included.

Supplementary Figure 5 Prognostic genes expression profiles in different normal tissues.

Supplementary Figure 6 Single-gene GSEA of four tumor clusters for KEGG pathway enrichment. GSEA, gene set enrichment analysis; KEGG, Kyoto Encyclopedia of Genes and Genomes; FC, fold change.

Supplementary Figure 7 The expression profile of **(A)** OXPHOS-associated and **(B)** glycolysis-associated genes for each cell. OXPHOS, oxidative phosphorylation.

Supplementary Figure 8 The expression profile of other key genes for each cell.
