# Supplementary figures and images for: Identification of Hub Genes Associated With Clear Cell Renal Cell Carcinoma by Integrated Bioinformatics Analysis
Source: Front Oncol. 2021 Sep 30;11:726655. doi: 10.3389/fonc.2021.726655 (PMC8516333; doi:10.3389/fonc.2021.726655)

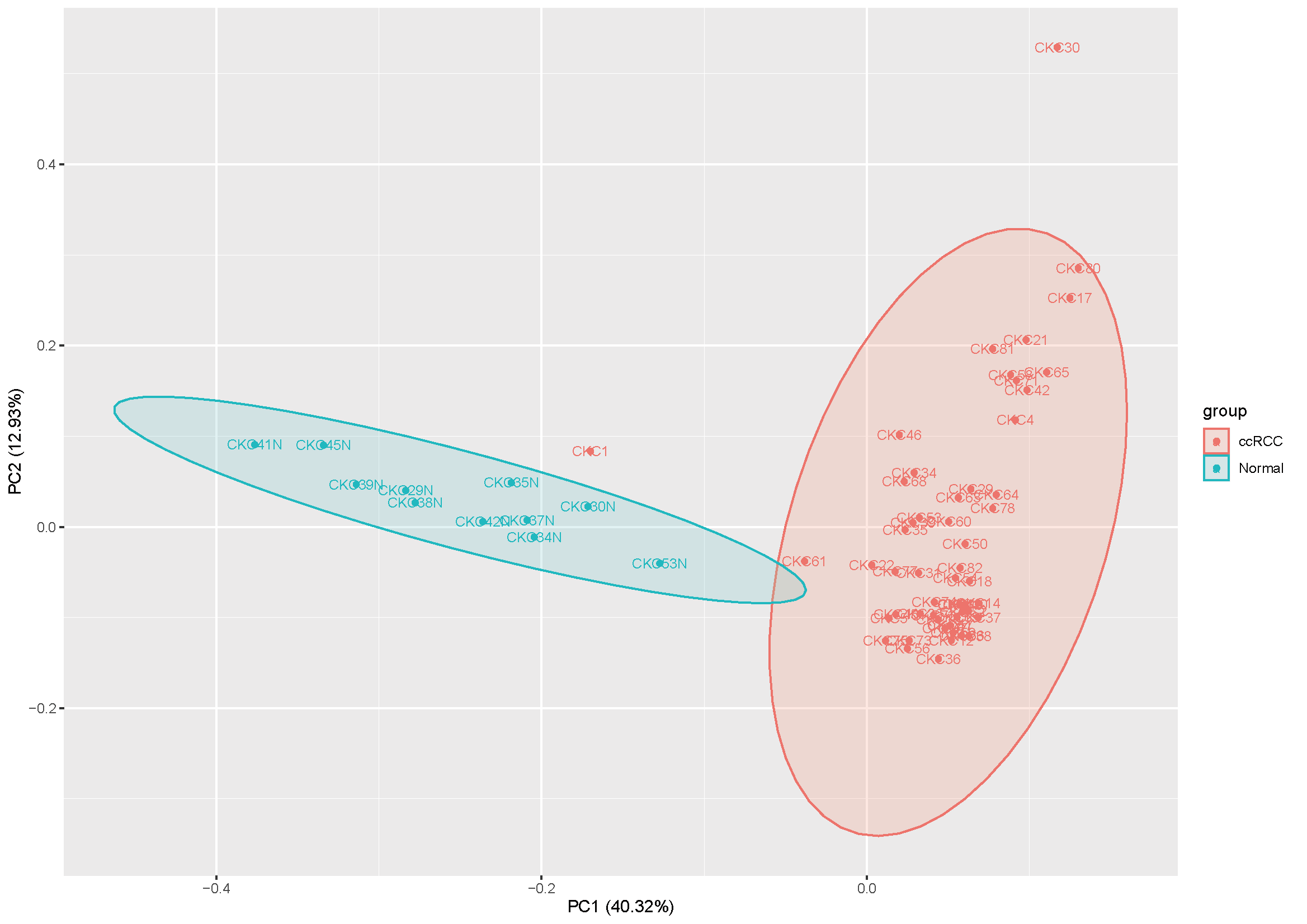

Supplement: Supplementary Figure 1 — PCA based on the whole gene list. PCA, principal component analysis. [file Image_1.tiff]

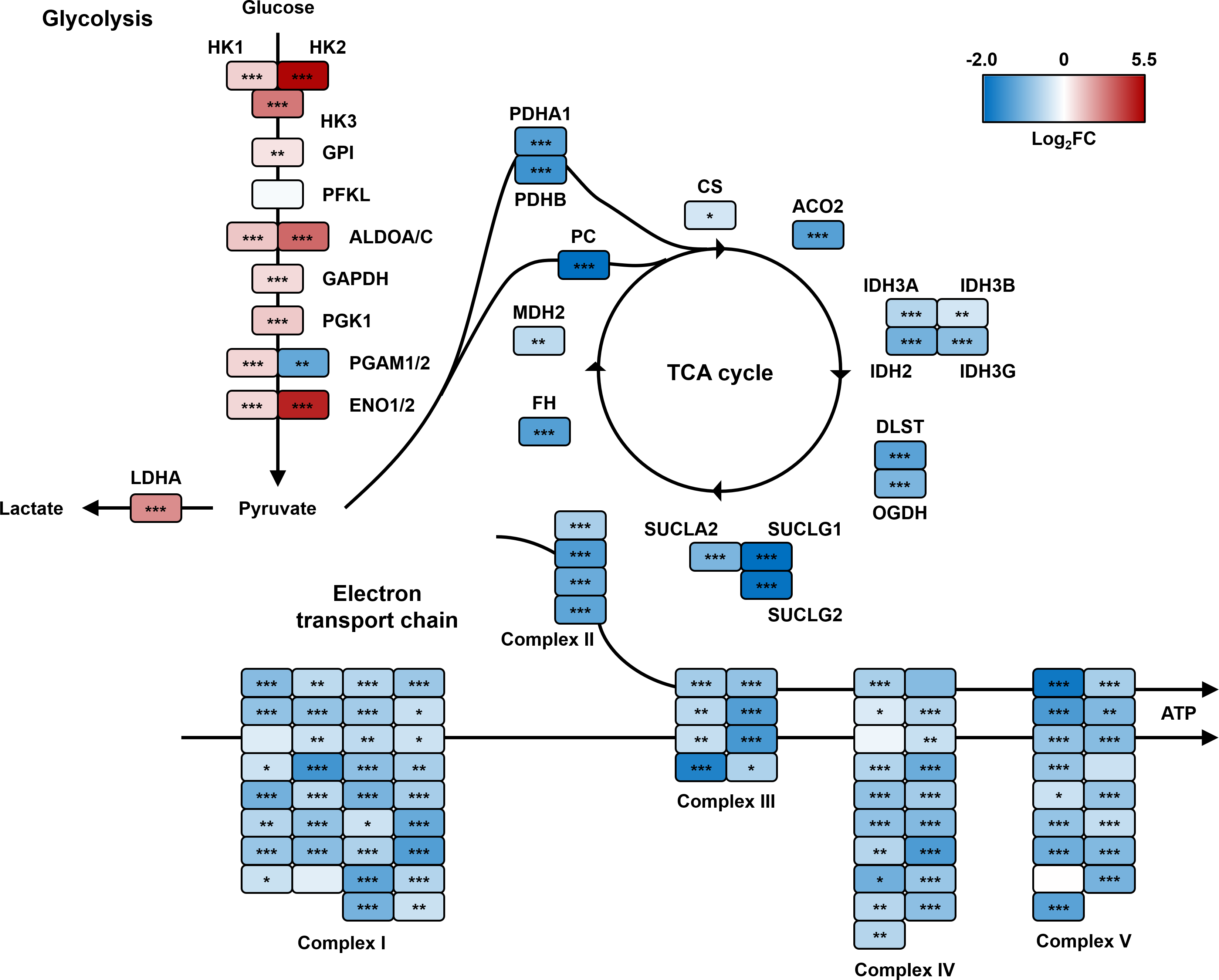

Supplement: Supplementary Figure 2 — Schematic of metabolic pathways and their selected genes for glycolysis, TCA and electron transport chain, with the log2FC level between ccRCC and control samples in the GSE126964 dataset. *p<0.05; **p<0.01; ***p<0.001. FC, fold change; ccRCC, clear cell renal cell carcinoma; TCA, tricarboxylic acid cycle. [file Image_2.tif]

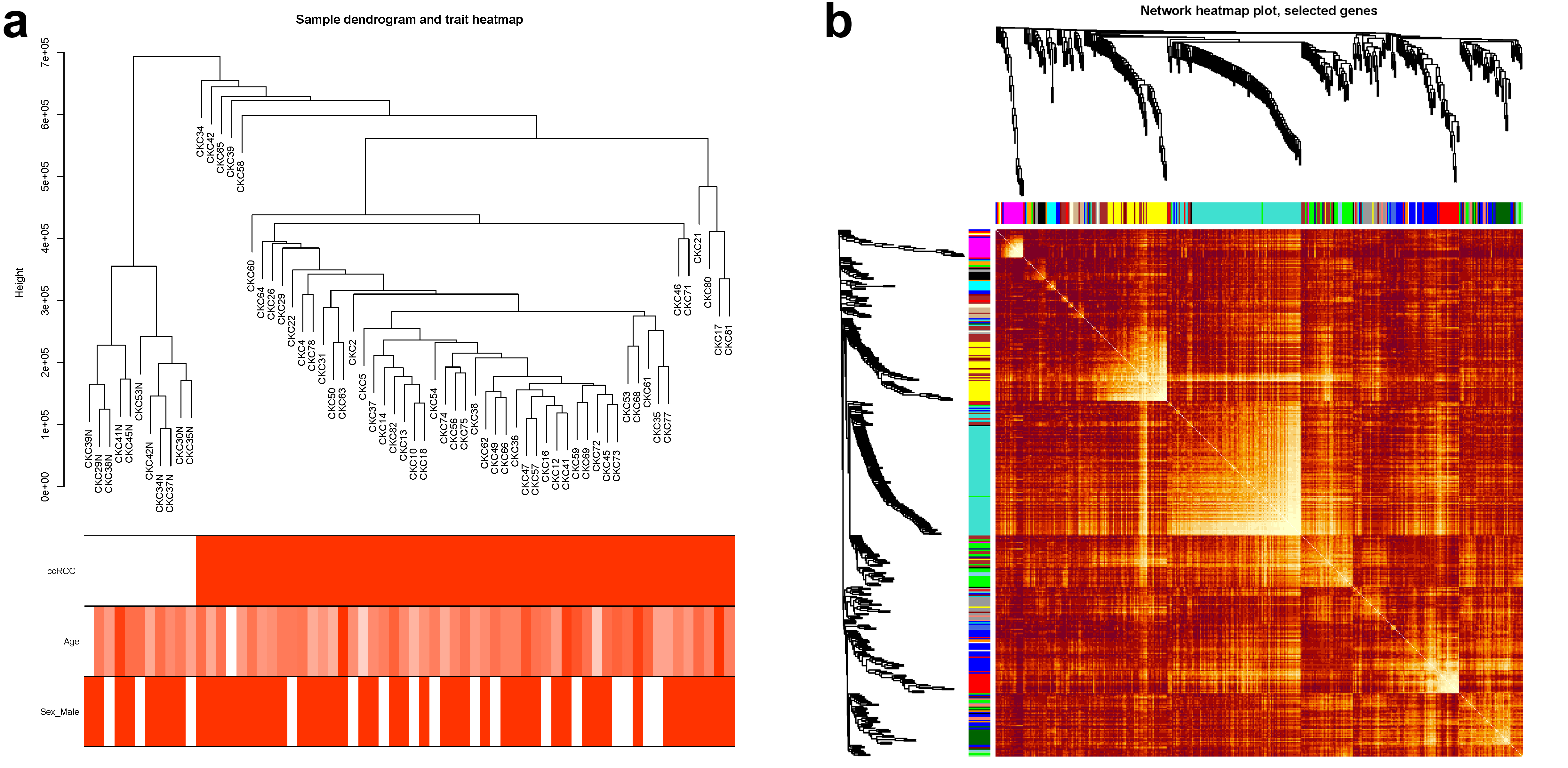

Supplement: Supplementary Figure 3 — (A) Sample clustering was conducted to detect outliers. All samples are located in the clusters and pass the cutoff thresholds after removing outliers. (B) Heatmap depicting the TOM of genes selected for weighted co-expression network analysis. The light color represents lower overlap, and red represents higher overlap. TOM, Topological Overlap Matrix. [file Image_3.jpeg]

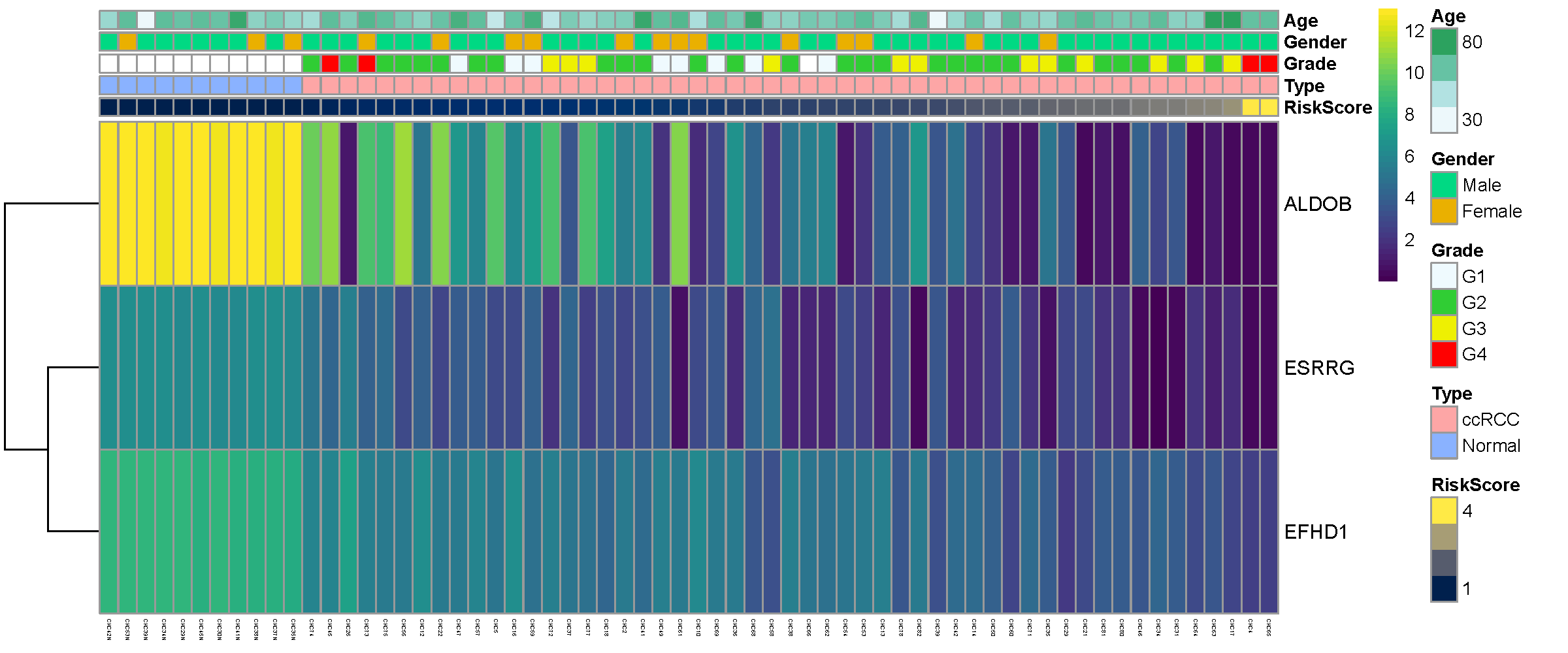

Supplement: Supplementary Figure 4 — The overall survival stratified by the high and low-risk score groups was plotted for the GSE126964 dataset. Detailed risk scores, survival information and heat maps of gene expression are also included. [file Image_4.tif]

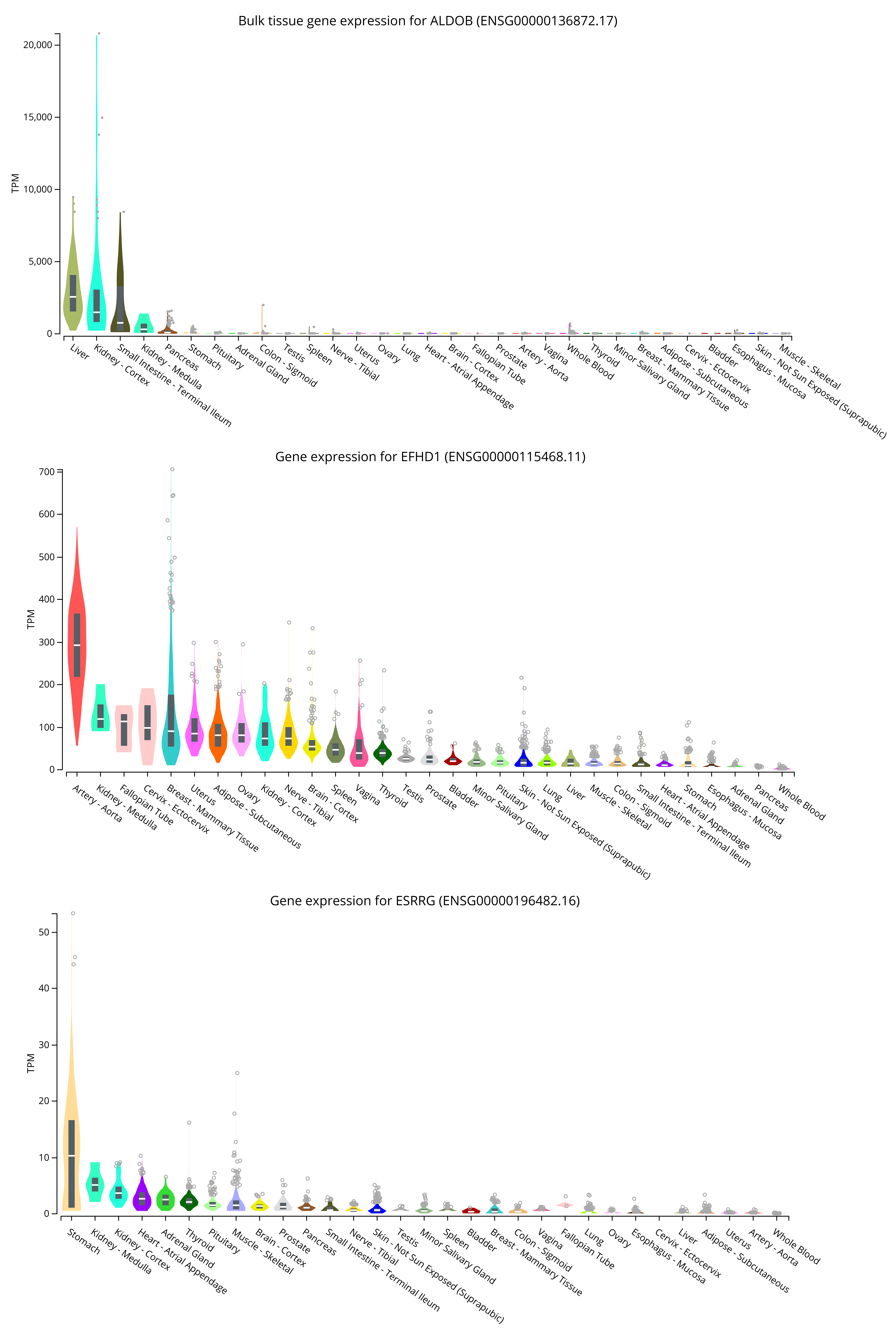

Supplement: Supplementary Figure 5 — Prognostic genes expression profiles in different normal tissues. [file Image_5.tif]

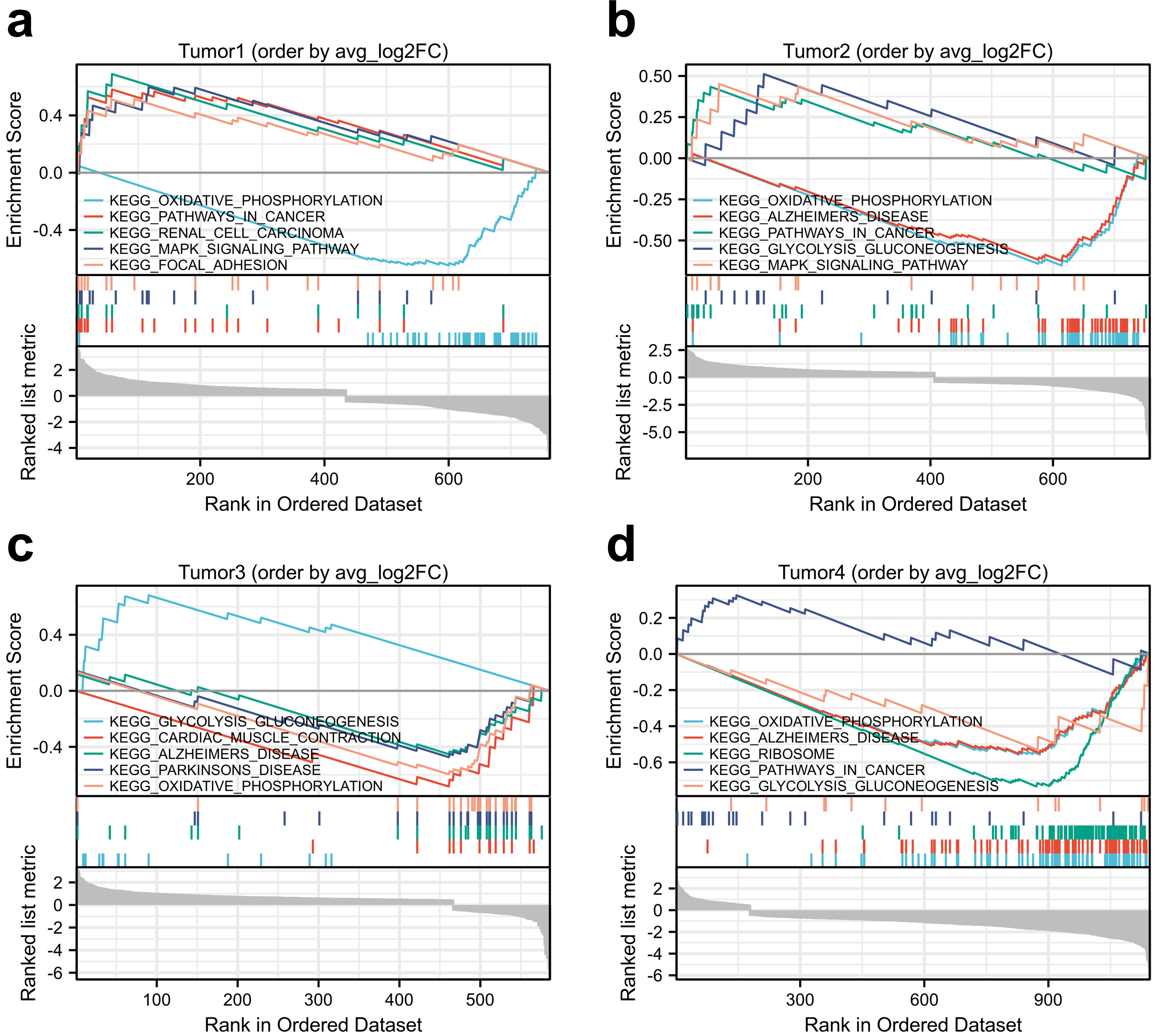

Supplement: Supplementary Figure 6 — Single-gene GSEA of four tumor clusters for KEGG pathway enrichment. GSEA, gene set enrichment analysis; KEGG, Kyoto Encyclopedia of Genes and Genomes; FC, fold change. [file Image_6.jpeg]

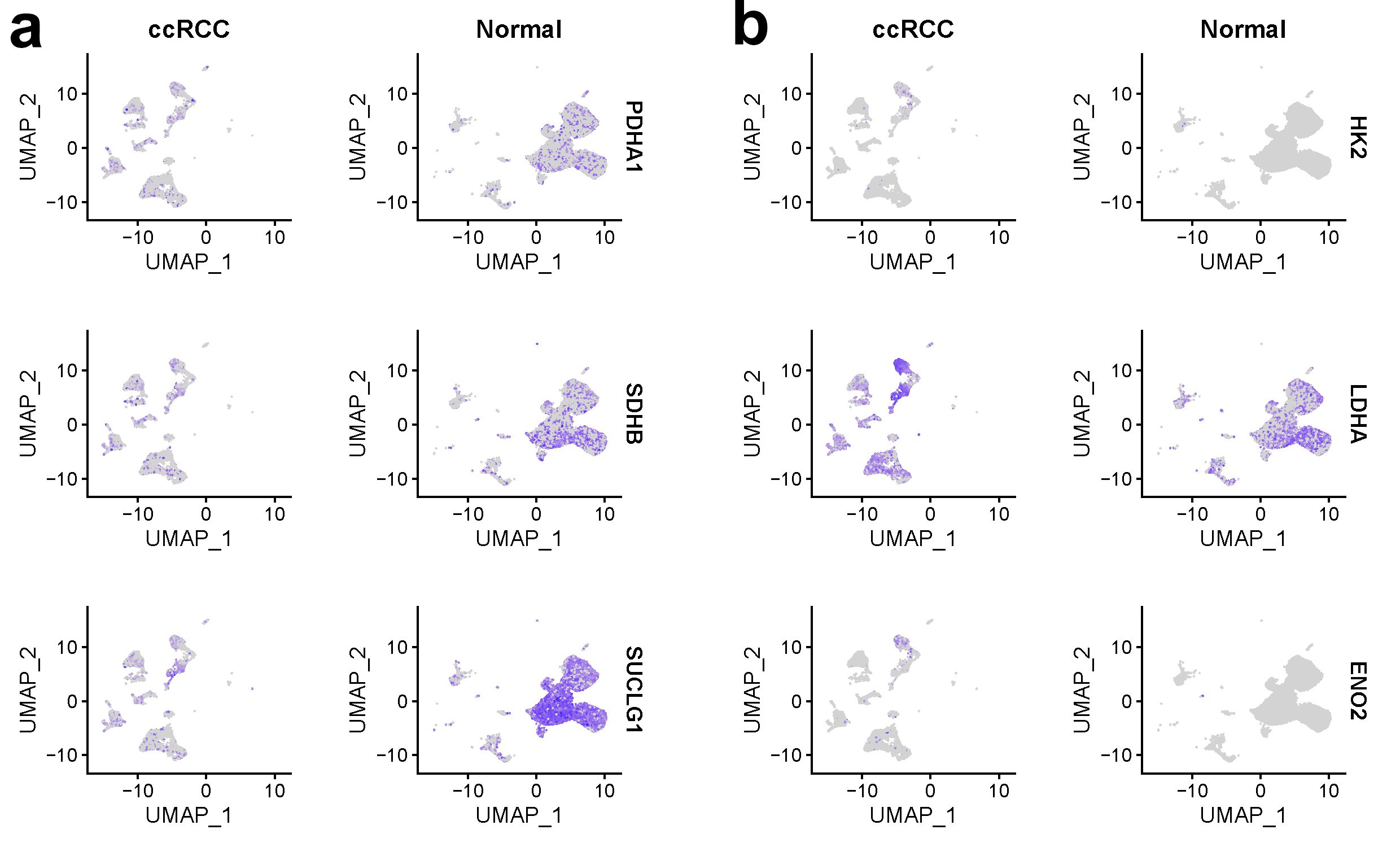

Supplement: Supplementary Figure 7 — The expression profile of (A) OXPHOS-associated and (B) glycolysis-associated genes for each cell. OXPHOS, oxidative phosphorylation. [file Image_7.tif]

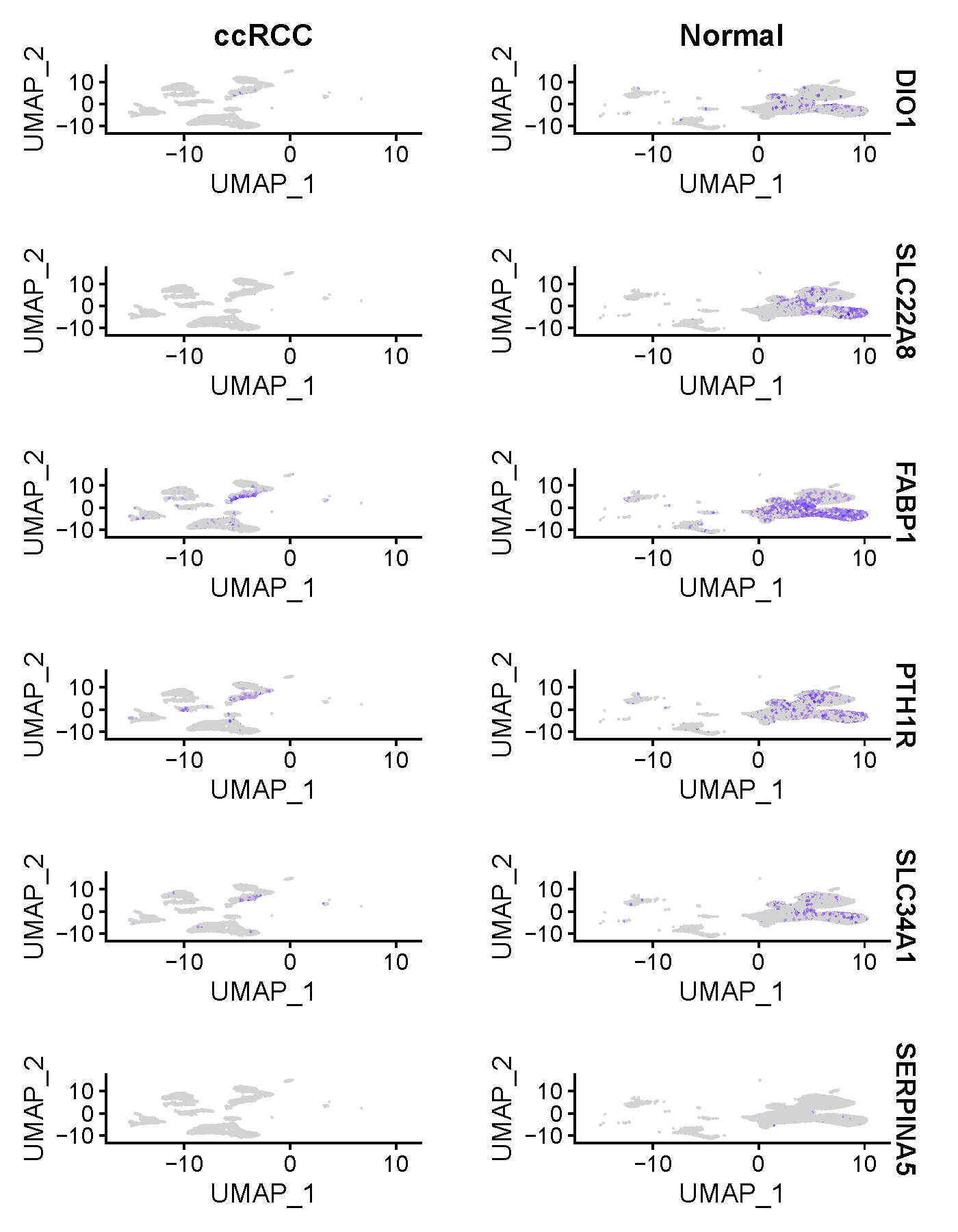

Supplement: Supplementary Figure 8 — The expression profile of other key genes for each cell. [file Image_8.tif]
